# Everybody's Page

**Published:** 1891-06-13

**Authors:** 


### Everybody's Page.

The total number of deaths in Austria from November, 1889, to March, 1890, directly attributable to influenza, was 2,823.

Professor Uffelman, of Rostock, has written a very complete and clever manual of the domestic hygiene of the child; he takes the child from birth to adolescence, considering it from a hygienic standpoint. The manual has been translated, and is being published by Putnam.

Berlin medical men object to their carriages being kept waiting in the crowded streets, and so in future their coachmen are to don white hats, which will distinguish them and show their right of way. And in a short time we may expect to hear that the number of doctors' carriages in Berlin is considerably on the increase.

Though the two-eyed spectacle which is such a common sight in Germany, even amongst children, cannot claim a greater antiquity than six hundred years, the use of glasses for the sight can be traced back to a period two thousand years ago. The Emperor Nero, who was short sighted, was one of the ancients who made use of a glass. He is said to have used a concave one to better watch the gladiators, and no doubt this enabled him to enjoy a much clearer view of the burning of the divine city than he would otherwise have enjoyed, though we are not aware of any historical statement vouching for the fact that he used a glass on that memorable occasion.

Mr. Sidney V. Lowell relates in the *Brooklyn Medical Journal* a curious instance of the phenomenon that some persons who have had part of a limb cut away subsequently have feelings so closely resembling those which they had in the lost member before its separation that the impression on the mind is precisely the same as if that part of the body were still present. An officer lost his leg, which after removal was left on the ground in a wild and remote region. The weather that followed was bitterly cold, and the officer complained that his leg was freezing. A brother officer went to the place where the limb had been left and buried it, after which the maimed man ceased to have any unpleasant sensations.

The desirability of altering the law of punishments so as to allow of sentences intermediate between two years, which is the maximum sentence of ordinary imprisonment, and five years, the minimum of penal servitude, has at length become so generally recognised that there is every probability of a speedy change in the law. We have sufficient charges to bear without needlessly saddling ourselves with additional and quite unnecessary ones by keeping prisoners in confinement longer than is necessary. But there are higher and more weighty motives for a change than a mere monetary one. Every year to which a prisoner is sentenced beyond the necessity of the case entails unjustifiable suffering, not only on the part of the prisoner, but on his innocent family who are affected equally with if not more than himself by the disgrace, and are subjected to the discomfort arising from being deprived of the advantages of his labour and protection. It seems sometimes to be forgotten that the object of punishment is to deter, not to degrade and cause undue misery. Why should the judge who assigns sentences intended to cure moral evils be precluded from using discretion? We do not fetter the doctor with hard and fast rules as to the doses which he administers to his patients.

During the recent epidemic of influenza in Chicago, immense quantities of Phenacetine were prescribed, this seeming to be the favourite remedy, and supplanting antipyrine.

To most people in this country the Indian mango is known only in the form of chutnee. In India, where it is valued as highly as the peach is with us and has as many varieties as the apple, it forms a considerable portion of the food of large classes of the native inhabitants, who out of the thick juice make thin cakes known as *amsatta*. Like our mushroom tribe, however, every mango is not wholesome, and the horse mango, which is largely eaten by the poorer classes, often produces cholera and dysentery.

The officers of the Society for the Prevention of Cruelty to Animals have done something for the welfare of horses on race days. We well remember in the days of our youth seeing about eight good men sitting behind a small pony on their way to Epsom, and in the hill by the Durdans the pony would sit down from sheer inability to move its legs. But now things are altered, and the good and bad man alike has to be merciful to his beast. The general condition of draught horses at Epsom races this year was reported to be excellent.

If one man more than another ought to suffer from perplexity just now it is he who is tormented by attacks of rheumatism, for if he is a sensible man he has laid out much money on Jaeger clothing, he has slept in Jaeger sheets, and has dried his tears with a Jaeger pocket-handkerchief. But now the advocates of the "hygienic excellence of Irish linen" have arrived upon the scene; they tell us of men who for sixty years have swathed themselves in wool and have not reaped the faintest benefit, and who on taking to wearing *real* Irish linen next their skin have been freed from rheumatism for ever! We are told that linen is invaluable in every form of skin disease, and we are asked how many of the six or seven thousand people in London recently swept off by diseases of the respiratory organs were clothed in pure flax linen. Are we going back to Hippocrates, who recommended the use of "pure fine linen next the skin"? But really the constant discovery of the last new thing in treatment is, to speak kindly, just a trifle bewildering.

It is none too soon that the subject of poor-law relief has attracted attention if the all-important question of how to deal satisfactorily with the dependent poor among us is to be solved in such a manner as to put a stop to the existence in our midst of a distinct race of paupers. The immigration of foreign destitute men and women does not help those who are striving to secure some kind of uniformity of administration of legal relief in this country. The Bishop of Bedford is eager for the abolition of casual wards; for his experience, like that of others who understand the question, is that it takes a little time only to change the man or woman who drops into a casual ward into the habitual casual. As things are a man can take his discharge from the house as soon as he pleases, and so long as this is permitted and the Guardians are not entrusted with power for longer detention and with greater discretion in dealing with applicants for relief, the evil will exist, and there will be a maximum of poor rates for a minimum of good effected.

